# Le genu recurvatum : une complication grave de l’injection intraquadricipitale en milieu africain

**DOI:** 10.48327/mtsi.v6i2.2026.813

**Published:** 2026-06-17

**Authors:** Michel ONIMUS, Anselme YAFONDO

**Affiliations:** 1 Faculté de médecine de Besançon, 19 Rue Ambroise Paré, 25000 Besançon, France; 2 Centre de rééducation pour handicapés moteurs (CRHAM) Bangui, République centrafricaine; 3 Service d’orthopédie-traumatologie, CHU Communautaire, Bangui, République centrafricaine Auteur correspondant : mpf.onimus@gmail.com

**Keywords:** Genu recurvatum, Injection intraquadricipitale, Quinine, Chirurgie, République centrafricaine, Afrique subsaharienne, Genu recurvatum, Intra-quadriceps injection, Quinine, Surgery, Central African Republic, Sub-Saharan Africa

## Abstract

**Introduction:**

Le genu recurvatum est une grave complication de l’injection intraquadricipitale. Matériel et méthode. Ce travail rapporte une série de 25 cas colligés lors de 104 missions de chirurgie orthopédique infantile réalisées en République centrafricaine, parmi lesquels 12 cas ont pu être revus avec un recul suffisant et/ou un bilan radiographique.

**Résultats:**

Le genu recurvatum handicape la marche et rend la course impossible; il devient gravissime quand il est bilatéral et quand il dépasse 90°. Les possibilités thérapeutiques sont limitées en raison des remaniements ostéo-articulaires se produisant au cours de la croissance avec hypoplasie de la trochlée, aplatissement de la partie antérieure des condyles fémoraux, subluxation antérieure du tibia. Malgré une bonne correction du recurvatum obtenue en postopératoire immédiat, le genou était raide en extension dans tous les cas revus sauf un.

**Conclusion:**

Les injections intraquadricipitales sont heureusement proscrites actuellement et cette complication devrait devenir exceptionnelle.

## Abstract (en)

**Introduction:**

Genu recurvatum is a serious complication of intra-quadriceps injections.

**Materials and methods:**

This study reports on 25 cases of genu recurvatum collected during 104 pediatric orthopedic surgery missions in the Central African Republic. Of these cases, 12 could be reviewed with sufficient follow-up and/or radiographic evaluation.

**Results:**

Genu recurvatum impairs walking and makes running impossible. It becomes extremely severe when it is bilateral or exceeds 90°. Treatment options are limited due to osteoarticular changes that occur during growth. These changes include trochlear hypoplasia, flattening of the anterior aspect of the femoral condyles, and anterior subluxation of the tibia. Despite the fact that the genu recurvatum was corrected immediately postoperatively, the knee was stiff in extension in all but one of the reviewed cases.

**Conclusion:**

Fortunately, intra-quadriceps injections are currently prohibited, so this complication should become rare.

**Figure:**
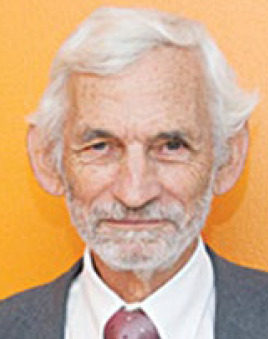

## Introduction

Le genu recurvatum peut présenter différents degrés de gravité, depuis la simple hyperextension du genou jusqu’au recurvatum de plus de 90°. À côté du genu recurvatum congénital, il peut être syndromique (notamment en cas d’arthrogrypose multiple congénitale, de myéloméningocèle ou de maladie de Larsen), ou paralytique, en cas de séquelles de poliomyélite antérieure aigüe. En milieu africain, l’étiologie la plus fréquente est le genu recurvatum secondaire à une injection intraquadricipitale de sels de quinine (traitement habituel de la poussée de paludisme chez l’enfant, actuellement proscrit mais encore souvent pratiqué car disponible partout, simple et peu onéreux). Ce travail analyse les aspects cliniques et radiographiques de cette déformation ainsi que les possibilités thérapeutiques à partir d’une série de 25 cas de genu recurvatum apparus après une injection intraquadricipitale. Ces cas ont été observés lors de 104 missions de chirurgie orthopédique infantile réalisées en République centrafricaine; parmi eux, 12 cas ont pu être revus avec un recul suffisant et/ou un bilan radiogra-phique et font l’objet de ce travail.

## Matériel et méthode

Il s’agit d’une étude rétrospective réalisée à partir du matériel rassemblé entre 1984 et 2024. Les missions se sont déroulées à Bangui ainsi que dans diverses villes de province, notamment Berbérati, Bossangoa, Bangassou, Bria, Mongoumba, Alindao, Dékoa, M’Baïki, Bossembélé, Bagandou… Un total de 30 cas de déformations du genou en recurvatum ont été observés, se répartissant en:

- deux cas de genu recurvatum sur arthrogrypose chez des patients âgés de 2 mois et 20 ans;
- trois cas de genu recurvatum congénital observés à l’âge de 2 mois, 7 mois et 20 ans;
- vingt-cinq cas de genu recurvatum apparus après une ou plusieurs injections intraquadricipitales de sels de quinine.

L’injection de sels de quinine a été incriminée dans tous les cas par les familles, qui précisaient qu’elle avait été pratiquée en raison de la survenue d’une fièvre importante. Les cas se répartissent en 16 filles et 9 garçons. Les enfants ont été vus à un âge moyen de 11 ans (extrêmes 6 ans - 18 ans). Le recurvatum moyen était de 52° en charge (extrêmes 30° - 100°); il était réductible en décubitus à 21° (extrêmes 0° - 40°) et bilatéral dans deux cas. L’âge auquel l’injection a été pratiquée était difficile à faire préciser par les familles, qui se contentaient de dire « Il y a longtemps… ». Les cas avec recurvatum inférieur à 30° ont été éliminés car n’entraînant que peu de retentissement sur la marche.

Les parents ont été informés du déroulement du protocole chirurgical et de la nécessité d’une longue rééducation. Tous les patients ont été opérés par désinsertion du quadriceps selon Judet *et al.* [8], associée dans deux cas à une ostéotomie fémorale métaphysaire basse de raccourcissement-flexion.

En postopératoire, les patients ont été immobilisés avec une gouttière plâtrée en position de flexion maximum. La rééducation a été débutée dès le premier ou deuxième jour postopératoire avec ablation de la gouttière plâtrée, mobilisations quotidiennes passives puis actives, et remise de l’attelle maintenant le genou en flexion maximum pour la nuit. La marche a débuté dès le 4^e^ ou 5^e^ jour dans des barres parallèles.

La plupart des cas n’ont pu être évalués que cliniquement, car, du fait des conditions dans lesquelles les consultations ont été réalisées, huit cas seulement ont pu bénéficier d’une radiographie.

## Résultats

Le recurvatum a été corrigé dans tous les cas. En postopératoire immédiat, la flexion du genou opéré était variable, de 50° à 130° (moyenne 105°), soit une correction moyenne de 126°.

Neuf cas ont pu être revus avec un recul de 6 mois à 10 ans. Dans tous ces cas sauf un, le recurvatum était corrigé mais le genou était soit totalement raide en extension (6 cas), soit avec une amplitude articulaire en flexion ne dépassant pas 10° (2 cas). Un seul cas bilatéral présentait une amplitude de flexion de 50° et 40°, cependant peu fonctionnelle. Une radiographie a pu être réalisée dans 8 cas, 4 fois en préopératoire (âge moyen 13 ans), 4 fois en postopératoire lors de la revue des patients (âge moyen 18 ans).

Il existait dans tous les cas des remaniements de l’extrémité inférieure du fémur, similaires en pré et en postopératoire, avec à des degrés variables une hypoplasie de la trochlée, une subluxation antérieure du tibia et/ou un aplatissement de la zone portante des condyles fémoraux.

En postopératoire immédiat, le recurvatum a été totalement corrigé dans tous les cas, passant de 21° en moyenne en décubitus à une flexion moyenne de 105°, donc dépassant l’angle droit. Ainsi, l’amplitude moyenne de correction était de 126°. Cependant, la flexion du genou était obtenue en peropératoire avec une sensation de ressaut traduisant la disparition de l’arrondi condylien. En postopératoire, l’attelle était retirée durant la journée, permettant les mobilisations et la mise en extension, mais la remise en flexion était très douloureuse et finalement abandonnée, et au 6^e^ mois postopératoire, le genou était totalement enraidi en extension malgré la qualité de la correction postopératoire immédiate et une rééducation immédiatement entreprise. Dans un seul cas bilatéral il persistait une amplitude de flexion de 50° et 40°. Malgré l’absence de flexion de leur genou, les patients revus se déclaraient satisfaits du résultat, car pouvant marcher sans aide et avec une boiterie peu marquée.

## Discussion

Ces missions de chirurgie orthopédique infantile ont été réalisées dans le cadre des activités de l’association des Amis comtois des missions centrafricaines (ACMC), ONG qui assure le soutien logistique ainsi que le lien avec le ministère de la Santé publique de la République centrafricaine. Plusieurs patients ont été opérés en province, dans différentes localités du pays. Jusqu’à une date récente, les radiographies n’étaient possibles que dans la capitale, expliquant que la plupart des patients n’ait pas bénéficié de bilan radio-graphique. Par ailleurs, dans le souci de toujours chercher à diminuer au maximum les coûts pour les familles, nous avons souvent (trop souvent…) renoncé à demander des radiographies pourtant très utiles, au moins au plan académique.

L’éloignement géographique explique que beaucoup d’opérés n’ont pas pu être revus : un tiers seulement l’a été, avec un recul variable de 6 mois à plusieurs années. Cependant, il semble possible de tirer des conclusions de l’analyse de la série en raison de la grande similitude clinique et radiologique observée dans les résultats documentés, rendant la série assez homogène.

L’étiologie de loin la plus fréquente des cas de genu recurvatum est l’injection intraquadricipitale de sels de quinine, qui représente 83 % des cas observés. Le genu recurvatum congénital est le plus souvent dépisté dès la naissance; le genu recurvatum sur arthrogrypose est rare, bien que l’arthrogrypose ne le soit pas en milieu africain. Bien que la notion d’une injection de sels de quinine ne repose que sur les déclarations des familles, cette étiologie semble pouvoir être retenue : jusqu’à une date récente, l’injection intraquadricipitale de sels de quinine était le traitement habituel de l’accès palustre chez l’enfant. La toxicité de la quinine est bien connue; l’injection provoque une fibrose du muscle qui entraîne habituellement une raideur complète du genou en extension [11,13]. Dans ces cas, de nombreuses publications ont montré que la chirurgie par désinsertion du quadriceps selon Judet ou la quadriceps-plastie selon Thompson [14] permettent, au prix d’une rééducation postopératoire bien conduite, une récupération notable de l’amplitude de flexion du genou, dépassant habituellement 90°. Selon la méta-analyse de Gutowski *et al.* [7] portant sur 33 articles incluant 797 patients opérés, les résultats de l’opération de Judet *et al*. sont comparables à ceux de la quadriceps plastie en termes de flexion du genou et de taux de complications, avec un déficit d’extension final un peu plus marqué avec l’opération de Thompson. Ainsi, dans la série de Fiogbé *et al*. portant sur 74 cas [5] opérés par quadriceps plastie, la flexion postopératoire moyenne est de 108°. Elle est de 125° dans la série de Sidibé *et al*. [12] portant sur 11 cas opérés par opération de Judet *et al.*, et de 140° dans la série de 20 cas de Muteti *et al*. [10] opérés par allongement du quadriceps. Gbenou *et al.* [6] retrouvent le recurvatum du genou secondaire à une injection dans 25 % des cas de leur série de 81 patients, Fiogbe *et al.* [5] dans 20 % des cas (sur 74 patients).

Les 25 cas de genu recurvatum rapportés ici ne représentent que 13 % de l’ensemble des 186 cas de séquelles d’injections intraquadricipitales observées au cours de nos missions chirurgicales. Ce nombre relativement faible s’explique par le fait que les recurvatum modérés, inférieurs à 30°, ont été éliminés.

**Figure 1:**
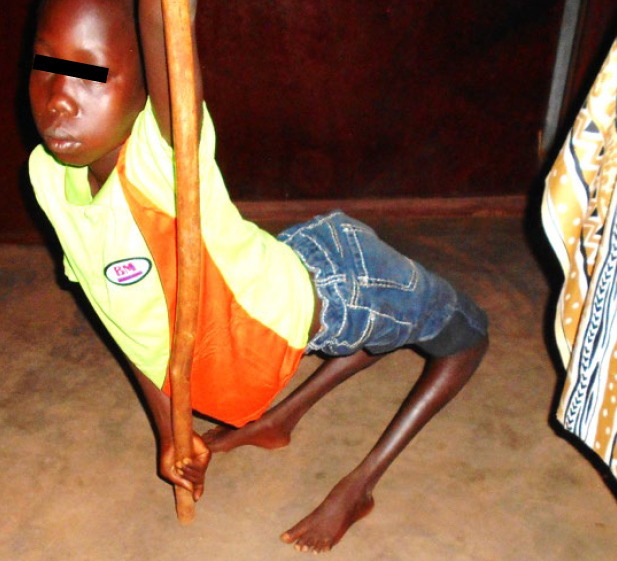
Genu recurvatum bilatéral de plus de 90°. L’enfant ne peut se déplacer qu’avec l’aide d’un bâton

Le recurvatum a été mesuré dans la position fonctionnelle, donc en charge. Son importance était variable, de 30° à 100°, entraînant un niveau de handicap également très variable. Lorsque le recurvatum est unilatéral et inférieur à 50°, la station debout est possible en appui monopodal sur le membre sain. La marche est possible, avec une boiterie accentuée, mais l’enfant ne peut pas courir. Quand le recurvatum est bilatéral et supérieur à 50°, la marche devient presque impossible. Un enfant présentait un recurvatum bilatéral de 90° et la station debout n’était possible qu’avec appui sur un bâton (Fig. 1). Dans tous les cas, le recurvatum était partiellement réductible, ramené à 21° en moyenne en décubitus. Dans 3 cas, il se réduisait presque totalement à moins de 10°.

La correction par désinsertion du quadriceps, décrite par Judet *et al*. pour la correction des raideurs post-traumatiques du genou [8], a également été proposée par Bell *et al*. [3] pour la correction du genu recurvatum congénital. Elle nécessite une désinsertion complète du quadriceps, réalisée par une longue incision latérale. Le geste est facile au niveau du vaste latéral et du crural, mais devient plus difficile au niveau de vaste médial : il faut désinsérer les fibres les plus médiales, en évitant des veines dont le saignement est difficile à contrôler. En pratique, il faut désinsérer le vaste latéral vers le haut jusqu’au niveau de la crête du vaste latéral. On peut alors soulever le muscle et progressivement désinsérer de haut en bas les fibres médiales. Lorsque les principales rétractions sont libérées, la flexion du genou peut se faire avec sensation de craquements qui traduisent la rupture des éléments rétractés résiduels. Il est nécessaire de s’aider d’une section de l’aponévrose de la face antérieure de la cuisse et d’une section du tendon du droit fémoral à son insertion sur l’épine iliaque antéro-inférieure. Lors de la mise du genou en flexion, le corps musculaire du quadriceps descend de plusieurs centimètres, traduisant le degré de rétraction du muscle. Cette flexion du genou se fait avec une flexion de la hanche, évitant un étirement du nerf fémoral.

**Figure 2:**
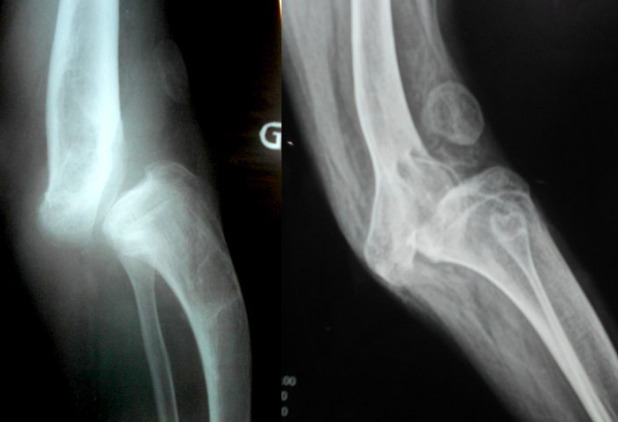
Importants remaniements ostéo-articulaires observés dans deux cas de genu recurvatum. Noter la quasi aplasie de la trochlée et la subluxation antérieure du tibia. Il s’agit presque d’une luxation dans l’observation de droite

**Figure 3:**
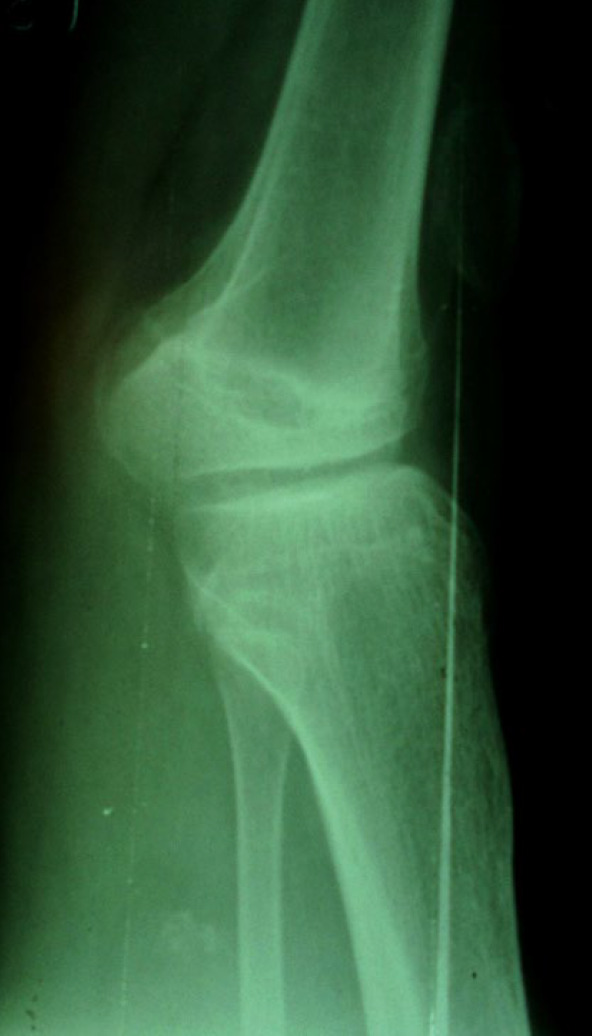
Séquelle d’injection intraquadricipitale. Le recurvatum est modéré mais le genou était totalement raide en raison de l’aplatissement de la surface portante des condyles

La détérioration du résultat immédiat s’explique par les modifications observées sur les radiographies. Ces modifications ont été retrouvées dans tous les cas où un bilan radiographique a pu être réalisé. On notait à des degrés variables une incurvation du fémur en recurvatum, une subluxation antérieure du tibia, une hypoplasie de la trochlée fémorale, et un aplatissement des condyles fémoraux avec perte de l’arrondi permettant la flexion. Les modifications les plus péjoratives étaient la subluxation antérieure du tibia et l’hypoplasie de la trochlée (Fig. 2). Dans deux cas, le remaniement principal était un aplatissement de la partie antérieure des condyles fémoraux et le genou était totalement raide en extension (Fig. 3).

**Figure 4:**
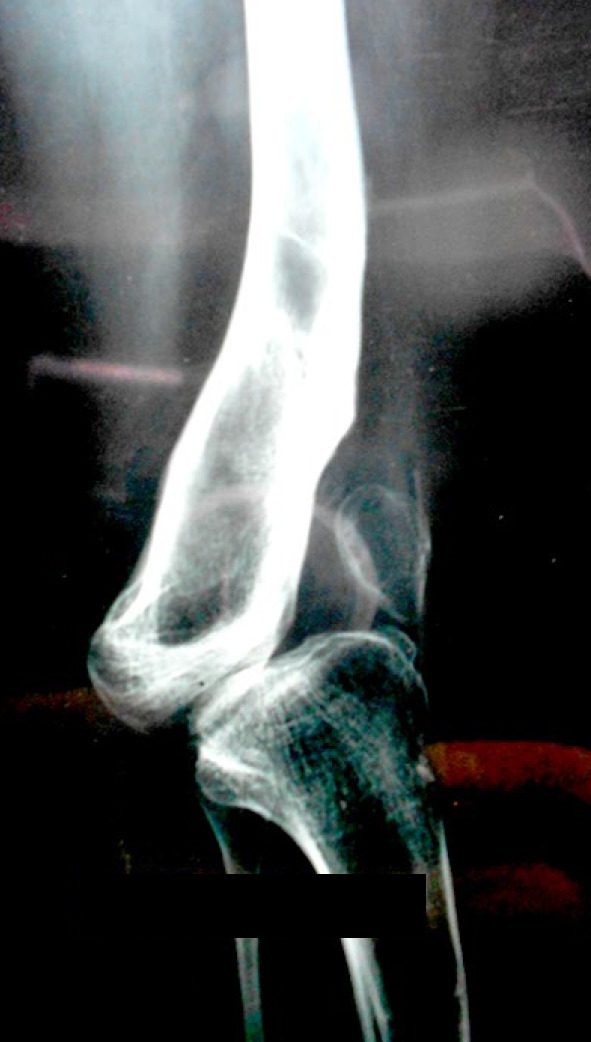
Radiographie prise 6 ans après une ostéotomie de flexion du fémur. Le recurvatum résiduel est compensé par la flexion fémorale, le tibia est subluxé en avant. La patiente se plaint de douleurs à la marche pouvant justifier la réalisation d’une arthrodèse du genou

Les aspects cliniques et radiographiques de cette série sont très comparables aux aspects décrits dans les rares observations de genu recurvatum congénital vus et traités tardivement [1,2,4,9]. Dans ces observations, les auteurs n’ont pas cherché à redonner une mobilité au genou. La correction du recurvatum a été obtenue par une ostéotomie fémorale métaphysaire inférieure de raccourcissement et de flexion, le tibia restant subluxé et articulé avec la trochlée fémorale plus qu’avec les condyles. Dans deux cas, la désinsertion du quadriceps n’a pas permis une correction satisfaisante du recurvatum et une ostéotomie complémentaire de raccourcissement-flexion a été réalisée. Dans ces deux cas, le recurvatum a été corrigé au recul, mais le genou était totalement raide en extension : son aspect radiographique était comparable aux données de la littérature, avec un tibia articulé avec la trochlée fémorale et subluxé en avant (Fig. 4).

## Conclusion

Les injections intraquadricipitales sont actuellement en règle générale proscrites. Elles restent encore trop souvent pratiquées, mais la fréquence de leurs complications est heureusement en baisse. L’apparition d’un genu recurvatum entraîne un handicap sévère, en particulier lorsqu’il est bilatéral. Il est, en partie seulement, amélioré par le traitement chirurgical qui permet seulement d’obtenir un genou raide en extension complète. Pour cette étude rétrospective présentant une technique déjà été décrite dans la littérature, l’avis d’un comité d’éthique n’a pas été sollicité.

## Source de financement

Ce travail n’a bénéficié d’aucune source de financement.

## Contributions des auteurs

MO : révision des dossiers et rédaction de l’article; YF : relecture de l’article et corrections.

## Déclaration de liens d’intérêts

Aucun lien d’intérêt n’a été déclaré.
