# Supplementary material for: Genu recurvatum: a serious complication of intra-quadriceps injections in the African context
Source: Med Trop Sante Int. 2026 Jun 17;6(2):mtsi.v6i2.2026.813. [Article in French] doi: 10.48327/mtsi.v6i2.2026.813 (PMC13423297; doi:10.48327/mtsi.v6i2.2026.813)
Supplement: Related texts

# Research article: Genu recurvatum: a serious complication of intra-quadriceps injections in the African context

## Introduction

Genu recurvatum can vary in severity, ranging from simple knee hyperextension to a bend of more than 90°. In addition to congenital genu recurvatum, the condition can be syndromic, particularly in cases of congenital multiple arthrogryposis, myelomeningocele, or Larsen’s disease. It can also be paralytic as a sequela of previous acute poliomyelitis. In Africa, the most common cause is genu recurvatum resulting from an intra-quadriceps injection of quinine salts, a standard treatment for acute malaria in children that is currently banned but still frequently used because it is widely available, simple, and inexpensive. This study analyzes the clinical and radiographic features of genu recurvatum, as well as treatment options, based on 25 cases that developed following an intra-quadriceps injection. The cases were observed during 104 pediatric orthopedic surgery missions in the Central African Republic. Among these cases, 12 could be reviewed with sufficient follow-up and/or radiographic evaluation, and they are the subject of this study.

## Materials and methods

This is a retrospective study based on data collected between 1984 and 2024. The missions took place in Bangui, as well as various provincial cities, including Berbérati, Bossangoa, Bangassou, Bria, Mongoumba, Alindao, Dékoa, M’Baïki, Bossembélé, and Bagandou.

A total of 30 cases of genu recurvatum deformities were observed. They are broken down as follows:

- Two cases of genu recurvatum associated with arthrogryposis in patients aged two months and 20 years.
- Three cases of congenital genu recurvatum were observed in patients aged two months, seven months, and 20 years.
- Twenty-five cases developed after one or more intra-quadriceps injections of quinine salts.

In all cases, the families blamed the quinine salt injection, stating that it was administered due to a high fever. There were 16 girls and 9 boys in the study. The children were examined at an average age of 11 years old (range 6–18 years old). The average recurvatum was 52° under load (range 30°–100°) and reducible to 21° in the supine position (range 0°–40°). It was also present bilaterally in two cases. The families had difficulty specifying the age at which the injection was administered, only saying, “A long time ago…” Cases with a recurvatum of less than 30° were excluded because they had little impact on walking ability.

Parents were informed of the surgical procedure and the need for a lengthy rehabilitation period. All patients underwent quadriceps detachment surgery according to Judet *et al.* [8], combined with a shortening-flexion osteotomy of the lower femoral metaphysis in two cases.

Postoperatively, patients were immobilized with a plaster splint in maximum flexion. Rehabilitation began on the first or second day after surgery with the removal of the splint, followed by daily passive and then active mobilization and the application of a brace to maintain maximum knee flexion overnight. Patients began walking as early as the fourth or fifth day using parallel bars.

Due to the conditions under which the follow-up visits were conducted, most cases could only be evaluated clinically, and only eight cases were able to undergo radiography.

## Results

The recurvatum was corrected in all cases. In the immediate postoperative period, flexion of the operated knee varied from 50° to 130° (mean 105°), representing an average correction of 126°. A total of nine cases were reviewed, with follow-ups ranging from six months to 10 years. The recurvatum was corrected in all but one case, though the knee was either completely stiff in extension (six cases) or had a flexion range of motion not exceeding 10° (two cases). One bilateral case presented with a range of motion of 50° and 40°, though it was not very functional. X-rays were obtained in eight cases: four were obtained preoperatively (mean age 13 years), and four were obtained postoperatively during patient follow-up (mean age 18 years).

There were remodeling changes at the distal end of the femur in all cases, with similar changes pre- and postoperatively. These changes included varying degrees of trochlear hypoplasia, anterior subluxation of the tibia, and/or flattening of the weight-bearing surface of the femoral condyles. During the immediate postoperative period, the recurvatum was fully corrected, changing from an average of 21° in the supine position to an average of 105° of flexion, exceeding a right angle. Thus, the average correction was 126°. However, knee flexion was achieved intraoperatively with a clicking sensation, indicating the disappearance of condylar rounding. Postoperatively, the splint was removed during the day to allow for mobilization and extension. However, returning to flexion was very painful and ultimately abandoned. By the sixth postoperative month, the knee was completely locked in extension, despite the quality of the immediate postoperative correction and rehabilitation that was initiated immediately. In a single bilateral case, a range of flexion of 50° and 40° persisted. Despite the lack of knee flexion, patients reported being satisfied with the outcome because they could walk without assistance and with only a slight limp.

## Discussion

These pediatric orthopedic surgery missions were carried out by the Association des Amis Comtois des Missions Centrafricaines (ACMC), a NGO that provides logistical support and serves as a liaison with the Central African Republic’s Ministry of Public Health. Several patients were operated on in various locations across the country, including the provinces. Until recently, X-rays were only available in the capital, which is why most patients did not undergo radiographic evaluation. Furthermore, to minimize costs for families, we often refrained from ordering X-rays, even though they are useful, at least from an academic standpoint.

Many of the patients who underwent surgery could not be followed up due to geographical distance: only one-third were followed up, with a follow-up period ranging from six months to several years. However, it is possible to draw conclusions from the analysis of the cases due to the high clinical and radiological similarity observed in the documented results, which makes the cases fairly homogeneous.

The most common cause of genu recurvatum is intra-quadriceps injection of quinine salts, accounting for 83% of observed cases. Congenital genu recurvatum is most often detected at birth. Genu recurvatum associated with arthrogryposis is rare, though arthrogryposis itself is not uncommon in the African context.

Although the idea of a quinine salt injection is based solely on family reports, this explanation seems plausible. Until recently, the standard treatment for malaria attacks in children was an intra-quadriceps injection of quinine salts. The toxicity of quinine is well known. The injection can cause muscle fibrosis, which usually results in complete knee stiffness in extension [11,13]. Numerous studies have shown that, in these cases, surgery involving quadriceps detachment according to Judet or quadriceps plasty according to Thompson [14] allows for significant recovery of knee flexion range with proper postoperative rehabilitation, usually exceeding 90°. According to the meta-analysis by Gutowski et al [7] of 33 articles involving 797 patients who underwent surgery, the results of the Judet *et al*. procedure are comparable to those of quadriceps plasty in terms of knee flexion and complication rates. However, there is a slightly more pronounced final extension deficit following the Thompson procedure. In the series by Fiogbé *et al.* involving 74 cases [5] treated with quadriceps plasty, the mean postoperative flexion is 108°. The mean postoperative flexion is 125° in the series by Sidibé *et al.* [12] involving 11 cases treated with the Judet *et al.* procedure and 140° in the series of 20 cases by Muteti *et al.* [10] treated with quadriceps lengthening. Gbenou *et al.* [6] found knee recurvatum secondary to an injection in 25% of their 81 patients, and Fiogbé *et al.* [5] found it in 20% of his 74 patients.

The 25 cases of genu recurvatum reported here represent only 13% of the 186 total cases of sequelae from intra-quadriceps injections observed during our surgical missions. This relatively low number is explained by the exclusion of cases with moderate recurvatum of less than 30°.

Genu recurvatum was measured in the functional position, i.e., under weight-bearing conditions. The severity of the condition varied from 30° to 100°, resulting in a similarly wide range of disability. With unilateral genu recurvatum of less than 50°, standing is possible with single-leg support on the unaffected limb. Walking is possible with a pronounced limp, but running is not. When the genu recurvatum is bilateral and greater than 50°, walking becomes nearly impossible. One child presented with a bilateral genu recurvatum of 90°, and standing was only possible with a cane for support (Fig. 1). In all cases, the genu recurvatum was partially reducible, reducing to an average of 21° in the supine position. In three cases, it reduced almost completely to less than 10°.

**Figure 1:** Bilateral genu recurvatum exceeding 90°. The child can only move with the aid of a cane

Correction via quadriceps detachment, which was originally described by Judet *et al*. for treating post-traumatic knee stiffness [8], has also been proposed by Bell *et al.* [3] for treating congenital genu recurvatum. This procedure requires complete detachment of the quadriceps via a long lateral incision. The procedure is straightforward for the vastus lateralis and vastus medialis but becomes more difficult for the vastus medialis because the most medial fibers must be detached. Care must be taken to avoid veins where bleeding is difficult to control. In practice, the vastus lateralis must be detached upward to the level of the vastus lateralis crest. Then, the muscle can be lifted and the medial fibers gradually detached from top to bottom. Once the main retractions are released, the knee can be flexed with a cracking sensation, which indicates the rupture of residual retracted elements. A section of the fascia on the anterior aspect of the thigh and a section of the rectus femoris tendon at its insertion on the anterior inferior iliac spine must be performed. When the knee is flexed, the quadriceps muscle body descends several centimeters, indicating the degree of muscle retraction. Knee flexion is performed with hip flexion to avoid stretching the femoral nerve.

The deterioration in the immediate outcome is explained by the changes observed on X-rays. These changes were found in all cases where an X-ray evaluation could be performed. To varying degrees, we noted a recurvatum curvature of the femur, anterior subluxation of the tibia, hypoplasia of the femoral trochlea, and flattening of the femoral condyles with loss of the rounded contour necessary for flexion. The most detrimental changes were anterior subluxation of the tibia and hypoplasia of the trochlea (Fig. 2). In two cases, the primary deformity was flattening of the anterior aspect of the femoral condyles, and the knee was completely stiff in extension (Fig. 3). The clinical and radiographic features of this series are very similar to those described in the rare reports of congenital genu recurvatum diagnosed and treated late [1,2,4,9]. In these reports, the authors did not attempt to restore knee mobility. Correction of the recurvatum was achieved by a shortening and flexion osteotomy of the lower femoral metaphysis, with the tibia remaining subluxed and articulating with the femoral trochlea rather than with the condyles. In two cases, quadriceps detachment did not allow for satisfactory correction of the recurvatum, and a supplementary shortening-flexion osteotomy was performed. In these two cases, the recurvatum was corrected retrospectively, but the knee was completely stiff in extension: its radiographic appearance was comparable to data in the literature, with the tibia articulating with the femoral trochlea and subluxated anteriorly (Fig. 4).

**Figure 2:** Significant osteoarticular changes observed in two cases of genu recurvatum. Note the near-total aplasia of the trochlea and the anterior subluxation of the tibia. The condition is nearly a dislocation in the right-hand image

**Figure 3:** Sequelae of an intra-quadriceps injection. The genu recurvatum is moderate, but the knee was completely stiff due to the flattening of the weight-bearing surface of the condyles

**Figure 4:** X-ray taken six years after a femoral flexion osteotomy. The residual recurvatum is compensated for by femoral flexion and anterior subluxation of the tibia. The patient complains of pain while walking, which may require knee arthrodesis

## Conclusion

Intra-quadriceps injections are currently generally contraindicated. They are still performed too often, but fortunately the frequency of their complications is decreasing. The development of genu recurvatum results in severe disability, particularly when it is bilateral. It is improved only partially by surgical treatment, which allows only for a stiff knee in full extension.

For this retrospective study presenting a technique already described in the literature, the opinion of an ethics committee was not sought.

## Funding

This work received no funding.

## Authors’ contributions

MO: review of medical records and drafting of the article; YF: proofreading of the article and corrections.

## Conflicts of interest

The authors declare no conflicts of interest.
